# Depression and Anxiety in Patients with Epilepsy, With or Without Other Chronic Disorders

**Published:** 2011-02-01

**Authors:** A A Asadi-Pooya, M R Sperling

**Affiliations:** 1 Department of Neurology, Shiraz University of Medical Sciences, Shiraz, Iran; 2 Jefferson Comprehensive Epilepsy Center, Department of Neurology, Thomas Jefferson University, Philadelphia, USA

**Keywords:** Anxiety, Chronic disorders, Depression, Epilepsy

## Abstract

**Background:**

Most people with epilepsy lead a normal emotional and cognitive life, however neurobehavioral problems can be found in a large number of patients. This study evaluates the prevalence of depression and anxiety among patients with epilepsy and determines whether having other chronic somatic illnesses increases the prevalence.

**Methods:**

Adults with epilepsy were recruited in either the inpatient epilepsy monitoring unit or the Outpatient Epilepsy Clinic at Thomas Jefferson University in 2006. Patients anonymously filled out a questionnaire, included data about age, sex, education, having other chronic illnesses, and degree of seizure control. The Hospital Anxiety and Depression scale was used to define the presence or absence of anxiety and depression.

**Results:**

Two hundreds patients participated, with a mean age of 40.3±16 years. Nineteen (9.5%) patients had depression and 49 (24.5%) had anxiety. Age, seizure control, and having other chronic illnesses did not have a significant relationship with either depression or anxiety. Gender was significantly related to anxiety, with females displaying greater frequency of anxiety than males. Depression was inversely related to education.

**Conclusions:**

It is probable that people with higher education use more effective ways to psychologically and physically adapt to their illness.

## Introduction

Most people with epilepsy have a normal emotional and cognitive life, however neurobehavioral problems can be found in a large number of patients. Higher rates of psychopathology have been reported in people with epilepsy compared with the general population, other neurological control groups, and with people with chronic non-neurological disorders.[1] Depression and anxiety are the most frequent types of psychiatric disorders identified in patients with epilepsy.[2] Some demographic and clinical variables such as gender and education may affect the prevalence of depression or anxiety.[3][4]

Depression and anxiety are also frequently found coexisting with other long-standing chronic medical conditions.[5] Nearly, half of asthma patients,[6] and many persons with arthritis,[7] cancer,[8] diabetes mellitus,[9][10] and cardiovascular disorders[11] suffer from depression and/or anxiety. The comorbidity of major depressive disorder or anxiety is associated with medical, psychiatric, and social problems. On the other hand, effective recognition and treatment of depression and anxiety may be associated with functional improvement in patients with comorbid medical disorders.[3]

It is well known that depression and anxiety are common among patients with epilepsy. However, factors that might increase the prevalence of depression or anxiety in patients with epilepsy are not determined well. The aim of this study was to evaluate the prevalence of depression and anxiety among patients with epilepsy referred to a tertiary center and to determine whether having other chronic somaticillnesses increases their prevalence. The secondary objective was to determine whether certain clinical and demographic variables, including age, gender, educational level and seizures control were related to the prevalence of depression or anxiety in these patients.

## Materials and Methods

In a cross-sectional questionnaire study, adults with a clinical diagnosis of epilepsy were recruited in either the inpatient epilepsy-monitoring unit or the outpatient epilepsy clinic at Thomas Jefferson University from September through December 2006. Patients aged 18 and above with a diagnosis of epilepsy for at least one year were eligible for this study. The diagnosis of epilepsy was made on clinical grounds by a board certified neurologist/epileptologist and all patients had to be taking anti-epileptic medications.

The questionnaires were filled out anonymously and included some details about gender, age, educational level (high school, some college, college graduate or higher), having other illnesses (other illness defined as any chronic somatic illness that requires continuing treatment), and degree of seizure control (presence or absence of seizures in the past six months). Complete seizure control was defined as no seizure of any type in the last 6 months prior to interview. TheHospital Anxiety and epression scale (HAD scale) was used to evaluate the anxiety level and depression in epilepsy patients. HAD scale was designed to assess symptoms of anxiety and depression in patients with somatic diseases.[12] The severe psychopathological symptoms and somatic symptoms of anxiety and depression were not included in the questionnaire. The HAD scale consisted of 14 items, measuring anxiety and depression on two separate subscales, each consisting of 7 items. There were four response categories, from 0 to 3 to each item with a maximum total for each subscale of 21. The low scores reflected absence of anxiety and/or depression, and > 10 score indicated a definite case on each subscale. In a number of studies, the HAD scale was shown to be reliable and valid in assessing anxiety and depression.[13][14][15]

Demographic variables and relevant clinical variables (i.e., age, gender, and educational level, seizure control, having depression or anxiety, and having chronic illnesses other than epilepsy) were summarized descript ely to characterize the study population. Statistical analyses were performed using Chi Square, and logistic regression to determine potentially significant associations, and a p value less than 0.05 was considered significant. In the first analysis, depression was the dependent variable, and age, gender, education, seizure control, and having other chronic illnesses were the independent variables. In the second analysis, anxiety was the dependent variable, and age, gender, education, seizure control, and having other chronic illnesses were the independent variables. This study was conducted with approval by the Thomas Jefferson University Institutional Review Board.

## Results

Two hundred patients filled out the questionnaires. The questionnaires were filled out anonymously, and about 15% of subjects were drawn from the Epilepsy Monitoring Unit and 85% from the Outpatient Epilepsy Clinic. Demographic and relevant clinical variables were summarized in Table 1.

**Table 1:** Demographic and clinical variables of patients studied^a^

**Age (years)**	**40.3 ± 16 (mean ± SD)**
Gender [Number (%)]	
Male	86 (43)
Female	112(56)
Education [Number (%)]	
High school or less	71 (35.5)
Some college	43 (21.5)
College graduate	83(41.5)
Seizure control [Number (%)]	
Completely controlled	60 (30)
Uncontrolled	133 (66.5)
Depression based on HAD scale score [Number (%)]	
Patients with depression	19 (9.5)
Patients without depression	181 (90.5)
Anxiety based on HAD scale score [Number (%)]	
Patients with anxiety	49 (24.5)
Patients without anxiety	151 (75.5)
Chronic illnesses other than epilepsy [Number (%)]	
Patients with other illness	58 (29)
Patients without other illness	141 (70.5)

^a^ Some data (less than 5.5 %) were missing in each category; therefore the sum in each category may be less than 200.

With logistic regression analysis, a significant relationship was observed between education and depression; they had an inverse relationship (p=0.02). Less educated patients were more often depressed than well-educated patients. Twelve out of 71 patients (16%) with high school education had depression, but just three in 83 patients (3%) with college graduate or higher educational level had depression. Age, gender, seizure control, and having other chronic illnesses were not related to having depression. With logistic regression analysis, a significant model that incorporated all the variables was not observed to predict anxiety (p=0.15). Age, education, seizure control, and having other chronic illnesses were not significantly related to having anxiety. However, subanalysis showed that anxiety was significantly related to gender within the model (p=0.013). Their relation was also significant with a separate analysis using Chi Square test (C2 = 6.2, p=0.01). Thirty-five out of 112 women (31%) had anxiety, but only 14 in 86 (16%) men reported anxiety. Fifty-eight patients (29%) reported having one or more chronic somatic disorders in addition to their epilepsy of which twelve patients had two or more other chronic disorders. Having multiple (two or more) chronic somatic disorders was not related to either anxiety or depression. The most common chronic disorders reported were high blood pressure (11 patients), asthma (9 patients), hypothyroidism (9 patients), diabetes (7 patients), hyperlipidemia (5 patients), arthritis (5 patients), autoimmnune disorders including systemic lupus erythematosus (3 patients), and cardiac disorders (3 patients).

## Discussion

In our study, the prevalence of anxiety among epilepsy patients was 24.5% and the prevalence of depression was 9.5%. The prevalence of anxiety among epilepsy patients is variable in different studies, from as low as 15% to as high as 25%.[5] The prevalence of depression among epilepsy patients is also variable in different studies, from as low as 9% to as high as 55%.[16][17][18][19] These various prevalences are possibly due to the different methodology in different studies including method of patient selection (e.g., hospital or community based) and the method of evaluation for depression and anxiety (different scales). The prevalence of depression and anxiety in patients with other chronic illnesses is also variable probably for the same reason,[5][6][7][8][9][10][11][20] however it is higher than the general population. Depression and anxiety have important implications in patients with other chronic illnesses. They may affect the quality of life, functioning, self-care behaviors, and consequently treatment success in patients with chronic disorders.[5][21] In addition, depressive and anxiety disorders assume an important role in the etiology, course, and outcomes associated with chronic diseases.[22][23] There is quite convincing evidence regarding relationship between diseases of the psyche and conditions affecting the cardiovascular system. This association is particularly strong in patients with depression and phobic anxiety. Depressive symptoms seem to be influential in predicting ischemic heart diseases. Phobic anxiety has also a clear association with increased risk of sudden cardiac death, which is probably related to increased susceptibility to arrhythmias. These associations with increased myocardial mortality have clinical therapeutic implications.[23] In one study,[24] it was observed that antidepressant drug adherence was associated with increased comorbid disease medication adherence and reduced total medical costs.

In this study, depression was more frequent among epilepsy patients with less education. In a prospective study,[4] predictive factors influencing the 2-year outcome of major depressive episodes were evaluated. Among the tested variables, anxious personality traits and a lower level of education predicted a high level of depressive symptoms. Individuals with higher levels of education may generally have greater cognitive resources, which in turn facilitate better coping strategies and adjustment to life with epilepsy. It is probable that people with higher education use more effective ways to psychologically and physically adapt to their illness and engage in more active coping strategies. Conversely, a low level of education may be a marker for a number of other underlying explanatory factors such as impaired cognition, that contribute to relatively poorer psychological adjustment.

This study has several limitations. One limitation is the collection of data from a tertiary epilepsy center. Therefore, these results may not be generalized. In addition, because the surveys were anonymously completed, certain information including seizure classification and seizure type, antiepileptic drugs or other drugs that might affect mood or anxiety are lacking. Finally, the relation between anxiety and gender needs further exploration, since there were a larger number of females compared with males in our study and also the logistic regression model was not significant.

In this study, having one or more chronic illness in addition to epilepsy did not increase the prevalence of depression or anxiety. The prevalence of depression and anxiety in patients with epilepsy, with and without other chronic somatic illnesses was not significantly different. However, the prevalence of depression and anxiety in patients with non-neurological chronic illnesses is higher than the general population.[25] The clinical implication of our study is that in epilepsy, anxiety and depression are more likely related to the endogenous factors such as neurochemical, electrophysiological and structural abnormalities in the CNS rather than the general disease burden.

Further community-based studies may address these issues. Effective recognition and treatment of anxiety and depression in patients with epilepsy may be associated with improved quality of life, better functioning, more beneficial self-care behaviors, and consequently treatment success in these patients.[21] In addition, attention to anxiety and depression may positively impact the economic burden of the comorbid disorders.[5] Therefore, recognition of risk factors that might increase the prevalence of anxiety or depression in patients with epilepsy is important.
